# Commentary: Editorial—Whole-body electromyostimulation: A training technology to improve health and performance in humans? volume II

**DOI:** 10.3389/fphys.2022.1028289

**Published:** 2022-10-17

**Authors:** Túlio Medina Dutra Oliveira, Marcelo Resende Machado, Marco Antonio Cavalcanti Garcia, Carla Malaguti

**Affiliations:** ^1^ Programa de Pós-Graduação em Ciências da Reabilitação e Desempenho Físico-Funcional, Universidade Federal de Juiz de Fora, Juiz de Fora, Brazil; ^2^ Universidade Salgado de Oliveira, Juiz de Fora, Brazil

**Keywords:** health-related outcomes, resistance exercise, functional limitations, WB-EMS, older people

We read with interest the recent study by Kemmler et al., 2022 reporting the feasibility and efficiency of whole-body electromyostimulation (WB-EMS) training for addressing important health-related outcomes in different populations such as healthy adults, people with low back pain, people with knee osteoarthritis, frail older people, and critically ill patients. We commend the authors for addressing this valuable training method that should be further studied due to its increasing popularity, time efficiency, joint-friendliness, and individualized application, which has increasingly been the subject of scientific research. Therefore, we intend to contribute to discussing the benefits of WB-EMS training specifically for older people.

Our recent systematic review (de Oliveira et al., 2022) compiled data on 283 older people aged 69 and 83 with metabolic syndrome, sarcopenic obesity, and sedentary lifestyles. Based on a subgroup analysis of our meta-analysis, it was possible to observe some differences in health-related outcomes between training with WB-EMS performed at different time points with or without the addition of protein supplementation, which is recommended by the European Working Group on Sarcopenia in Older People (EWGSOP) (Cruz-Jentoft et al., 2019).

The subgroup analysis revealed that in the short- (until 2 months) and medium-terms (two to 6 months), isolated training with WB-EMS did not affect habitual gait speed (SMD: 0.67, 95% CI: 0.02 to 1.36; n = 38) and handgrip strength (SMD: 0.52, 95% CI: 0.16 to 1.20; n = 38) outcomes. However, in the medium-term, the combination of WB-EMS training with protein supplementation led to moderate effects for the same habitual gait speed and handgrip strength outcomes (SMD: 0.68, 95% CI: 0.12 to 1.25; n = 85; SMD: 0.60, 95% CI: 0.19 to 1.00; n = 104, respectively). Indeed, protein supplementation may enhance resistance training results on measures related to strength and muscle hypertrophy (Kirwan et al., 2022). For instance, previous studies have shown that leucine intake during exercise stimulates the mammalian target of rapamycin complex 1 (mTORC1), which integrates anabolic signals for protein synthesis and avoids proteolysis in human skeletal muscles (Finger et al., 2014). On the other hand, appendicular muscle hypertrophy (SMD: 0.69, 95% CI: 0.30 to 1.09; n = 104) and isometric knee extension torque (SMD: 0.81, 95% CI: 0, 41 to 1.21; n = 106) were the outcomes that improved over the long-term (over 6 months) by isolated training with WB-EMS, with effect sizes ranging from moderate to large. These findings suggest that isolated training with WB-EMS, at least in older people, is promising when implemented in the long-term and, therefore, depends on good acceptability and adherence in this population. It is worth mentioning that the exercise protocols of the studies included for the analysis of these outcomes are very similar in terms of parameters, weekly frequency, intensity, dosage, and type of supplementation, which are reflected in the absence of indirectness and inconsistency, facilitating the interpretation of the results (de Oliveira et al., 2022).

Given these results, the short- and medium-term efficacy of WB-EMS without co-interventions in older people seems to be controversial, at least at present when the quantity and quality of evidence are low. For example, Bloeckl et al. (2022) found a significant short-term improvement in handgrip strength and performance in the Short Physical Performance Battery (SPPB), which assesses functional capacity (including the gait speed test) in a sample of frail older people who did not receive protein supplementation. The exercise protocol, current parameters, frequency, and duration of the WB-EMS session in Bloeckl et al. (2022) study were very similar to the studies included in our meta-analysis. Our systematic review suggests that adjuvant interventions to WB-EMS, such as protein supplementation, favored a significant change in outcome measures in a shorter time, such as handgrip strength and habitual gait speed in older people. Therefore, although effective, it is possible that the additional cost and multiple interventions can make adherence difficult.

As demonstrated in previous studies (Bloeckl et al., 2022; de Oliveira et al., 2022), WB-EMS has considerable potential to be an option for conventional training for older people who exercise below-recommended doses to impact strength and muscular hypertrophy positively. Moreover, WB-EMS seems to contribute to and overcome disabling conditions or functional limitations even due to reasons of time or motivation. Nevertheless, it is equally essential to consider establishing a nutritional plan parallel to resistance training to anticipate benefits in health-related outcomes, mainly when the long-term program is not feasible.

## References

[B1] Bloeckl J. Raps S. Weineck M. Kob R. Bertsch T. Kemmler W. (2022). Feasibility and safety of whole-body electromyostimulation in frail older people-a pilot trial. Front. Physiol. 13, 856681. 10.3389/fphys.2022.856681 35812334 PMC9263209

[B2] Cruz-Jentoft A. J. Bahat G. Bauer J. Boirie Y. Bruyère O. Cederholm T. (2019). Sarcopenia: Revised European consensus on definition and diagnosis. Age Ageing 48, 16–31. 10.1093/ageing/afy169 30312372 PMC6322506

[B3] de Oliveira T. M. D. Felício D. C. Filho J. E. Fonseca D. S. Durigan J. L. Q. Malaguti C. (2022). Effects of whole-body electromyostimulation on health indicators of older people: Systematic review and meta-analysis of randomized trials. J. Bodyw. Mov. Ther. 31, 134–145. 10.1016/j.jbmt.2022.03.010 35710211

[B4] Finger D. Goltz F. R. Umpierre D. Meyer E. Rosa L. H. Schneider C. D. (2014). Effects of protein supplementation in older adults undergoing resistance training: A systematic review and meta-analysis. Sports Med. 45, 245–255. 10.1007/s40279-014-0269-4 25355074

[B5] Kemmler W. Kleinöder H. Fröhlich M. (2022). Editorial: Whole-body electromyostimulation: A training technology to improve health and performance in humans? Volume II. Front. Physiol. 13, 972011. 10.3389/fphys.2022.972011 36111142 PMC9470239

[B6] Kirwan R. P. Mazidi M. García C. R. Lane K. E. Jafari A. Butler T. (2022). Protein interventions augment the effect of resistance exercise on appendicular lean mass and handgrip strength in older adults: A systematic review and meta-analysis of randomized controlled trials. Am. J. Clin. Nutr. 115, 897–913. 10.1093/ajcn/nqab355 34673936

